# The role of information and participation in overcoming users’ initial reluctance: a case study of a decentralized wastewater treatment plant

**DOI:** 10.3389/fpsyg.2024.1445320

**Published:** 2024-11-05

**Authors:** Sergio Vila-Tojo, Cristina Gómez-Román, Jose-Manuel Sabucedo

**Affiliations:** CRETUS, Department of Social Psychology, Basic Psychology and Methodology, Faculty of Psychology, University of Santiago de Compostela, Santiago de Compostela, Spain

**Keywords:** acceptance, decentralized wastewater treatment systems, psychosocial intervention, social perception, attitudes, priming

## Abstract

Decentralized wastewater treatment systems are a potential solution to the water crisis. However, in addition to advanced technology, successful implementation of these systems requires broad public willingness to use them. This paper presents the results of a three-phase psychosocial intervention with the users of a business building where a decentralized wastewater treatment plant was installed. The intervention, motivated by complaints from users due to their lack of knowledge about the existence of the plant, aimed at understanding and improving users’ perceptions of the building’s decentralized system. In the first phase, we conducted a focus group with a sample of workers (*n* = 6) to understand their knowledge and perception of the building’s decentralized wastewater treatment system. Once the main obstacles and facilitators were identified, we designed a second phase where a group of employees (*n* = 46) were exposed to environmental priming to improve attitudes toward the decentralized plant installed in the building. Finally, in the third phase, a bidirectional informative session was proposed, conducted by experts, to another group of workers (*n* = 25). Findings suggest that implementing specific psychosocial strategies, such as promoting environmental awareness and providing informative sessions, along with incorporating potential users throughout the process, contributes to better acceptance of the decentralized wastewater treatment plant. This work presents a real case in a pilot plant that can serve as a guide for addressing psychosocial resistance in similar projects.

## 1 Introduction

The installation of decentralized plants is proposed as a solution to the problem of water scarcity and water quality worldwide (Lens et al., 2005; Roefs et al., 2017). This type of system follows a circular economy principle for water reuse. It starts separating wastewater from the source, which allows the application of specific treatments that purify the water. Subsequently, the resulting water is reused for non-potable and potable purposes, depending on the treatment administered. However, despite the advantages and guarantees of this type of systems, people can be reluctant to use it, especially if they perceived any inconveniences in its installation or functioning (Brouwer et al., 2015; Ellis et al., 2021).

An example of this is the Porto do Molle Business Center in Nigrán, Galicia, Spain (case study), which accommodates 200–300 employees in coworking spaces for start-up companies. This building was, at that time, one of the few in Spain to operate with a decentralized system and, therefore, not rely on an external wastewater treatment plant for wastewater treatment. The building’s decentralized plant separates grey water from black water, which makes it possible, on the one hand, to reuse the water from the sinks for flushing the toilets. On the other hand, through anaerobic treatment of the sewage, two by-products are obtained: (a) nutrients for fertilizer production (Bisschops et al., 2019) and (b) biogas as an energy source (Hao et al., 2019).

Despite its innovative design and sustainable contribution to the building, most workers, especially new hires, were unaware of its existence. These workers became aware of the decentralized system following a technical problem that resulted in the emission of unpleasant odors. Although the problem did not compromise health, it caused significant discomfort among the workers who expressed concerns about the decentralized plant. Those negative perceptions toward the plant would have been caused by the high uncertainty associated with the lack of understanding of the technology. In this context, epistemic demand is activated, that is the need to seek information to resolve uncertainty (Sabucedo et al., 2020).

In order to reduce the uncertainty associated with the incident, we propose an intervention based on three theoretical axes: (a) knowledge, (b) trust and (c) participation. Following the foundations of the knowledge deficit model, providing information with the aim of increasing knowledge about the decentralized plant can contribute to reduce uncertainty and increasing positive attitudes (Bauer et al., 2007; Gustafson and Rice, 2016). However, considering that discomfort about the plant installation had already been expressed, simply increasing knowledge might not be sufficient (Schultz, 2002). In this sense, it is necessary to consider who and how the information is presented. The selection of trustworthy agents for the audience and the way in which the information is framed is a fundamental aspect for the message to be persuasive (Meyer, 1988). Moreover, it is necessary to actively involve workers (Wibeck, 2014) by facilitating a space that would allow them to openly express their concerns and generate a trust environment for greater permeability of the information provided.

On this basis, the intervention consisted of three sequential phases. In the first phase, a focus group explored workers’ knowledge and perceptions about the building’s decentralized wastewater treatment system. Based on the results of this initial approach, a second phase was carried out with another group of workers, who participated in an experiment using environmental priming to improve attitudes toward the decentralized treatment system installed in the building. Finally, we evaluated how a bidirectional informative session led by experts affected users’ perceptions of the decentralized plant.

## 2 Phase 1. Approaching workers’ perceptions of the plant

The decentralized plant at the business center is not the only case facing public resistance. In other jurisdictions, reuse projects have even failed due to this social rejection (Hurlimann and Dolnicar, 2010; Brouwer et al., 2015). For this reason, the starting point of the intervention in this building was to explore the extent to which psychosocial facilitators and barriers identified in other contexts were also present in this specific context (Gómez-Román et al., 2020; Hurlimann and Dolnicar, 2010; Mankad and Tapsuwan, 2011).

An unpleasant appearance or smell in the building, as in this case, can influence the formation of health risk perceptions (Etale et al., 2020; Fielding et al., 2018) and trigger negative emotional reactions related to fear or disgust that hinder acceptance, such as the well-known “yuck factor” (Po et al., 2003). This may lead people to reject decentralized plants if they perceive the system or the quality of the treated water as threatening their safety or that of their families (Fielding et al., 2018).

Beyond this relevant issue, other psychosocial factors can lead people to position themselves in favor or against these decentralized plants. Not being aware of a water scarcity problem may result in the perception that the decentralized plant is unnecessary, and thus reduce the level of acceptance (Fielding et al., 2018). Even recognizing the problem and the appropriateness of the plant to tackle it, people also consider other costs and benefits associated with decentralized plants before accepting it. The perception of high installation and maintenance costs could lead to low acceptance (Mankad, 2012); whereas the perception of high environmental and economic benefits would increase acceptance (Domènech and Saurí, 2010; Koetse, 2005).

It should be noted that the formation of perceptions and emotions is largely grounded in our social exchanges (Jaspal and Breakwell, 2014). When the proposal to reuse water comes from an in-group member, acceptance increases as there is greater credibility and trust between the actors (Ross et al., 2014; Schultz and Fielding, 2014). Moreover, if their use is perceived as a common practice (descriptive social norm) and approved (injuctive norm) by society, individuals are more likely to be inclined to adopt and endorse these systems (Lamichhane and Babcock, 2013; Liu et al., 2022).

In summary, different factors have been identified as influencing the acceptance of decentralized wastewater treatment systems. To find out whether these barriers and facilitating factors are also present in the building users, we designed a focus group with several workers to find out their reactions to the installed plant in the building.

### 2.1 Materials and methods

#### 2.1.1 Participants and procedure

The focus group included six building workers with different profiles: management, building, maintenance and housekeeping, cleaning, and building user companies. The group was composed of three men and three women, with ages ranging from 25 to 60 years. This variability allows us to obtain different perspectives and identify overlaps between the different profiles, which is valuable evidence contributing to the results’ validity (Bowen, 2008; Watts et al., 2017).

Our decision to limit the focus group to a maximum of six participants was guided by the recommendations of Krueger and Casey (1994) and Morgan (1997). This approach was chosen to maintain the quality of the discussion, as larger groups can lead to increased moderator intervention and participant inhibition, thereby compromising the richness of the data.

The focus group was conducted in a room within the building. Participants were invited to take part in a study focusing on the business center. Prior to starting, they were provided with a clear understanding of the study’s objective, privacy policy, and data protection measures. To ensure their full understanding and agreement, participants were asked to sign an informed consent form, thereby confirming their voluntary participation in the study.

An expert moderator facilitated the focus group session, which lasted approximately an hour and a half. The session followed a standardized script to gather accurate information (see Supplementary material). To start the conversation, the moderator began by asking about environmental concerns in the area. Then, questions were asked about participants’ knowledge of the water problem in the region and the building’s decentralized wastewater treatment technology. Participants were then asked to identify the potential advantages and disadvantages they believed could result from implementing this system and to evaluate their satisfaction with the decentralized wastewater treatment plant installed in the building.

#### 2.1.2 Data analysis

This work constitutes primary qualitative research, for which a thematic analysis approach was adopted (Thomas and Harden, 2008; Cruzes and Dyba, 2011) to identify, analyze, and report patterns (themes) in the data, coded segment by segment (Cruzes and Dyba, 2011). This means that each part of the text is individually examined to identify significant elements, which are then assigned codes that allow for systematic categorization and organization of the information. Through this coding process, it is possible to detect relevant patterns and themes throughout the discussion (Cruzes and Dyba, 2011). The thematic analysis of the focus group was conducted using Atlas.ti 9 software. The data analysis method is broken down into three phases: (1) coding, (2) enhancing coding reliability, and (3) synthesis. The process of each phase is detailed in Supplementary material.

### 2.2 Results and discussion of phase 1

**Figure 1:**
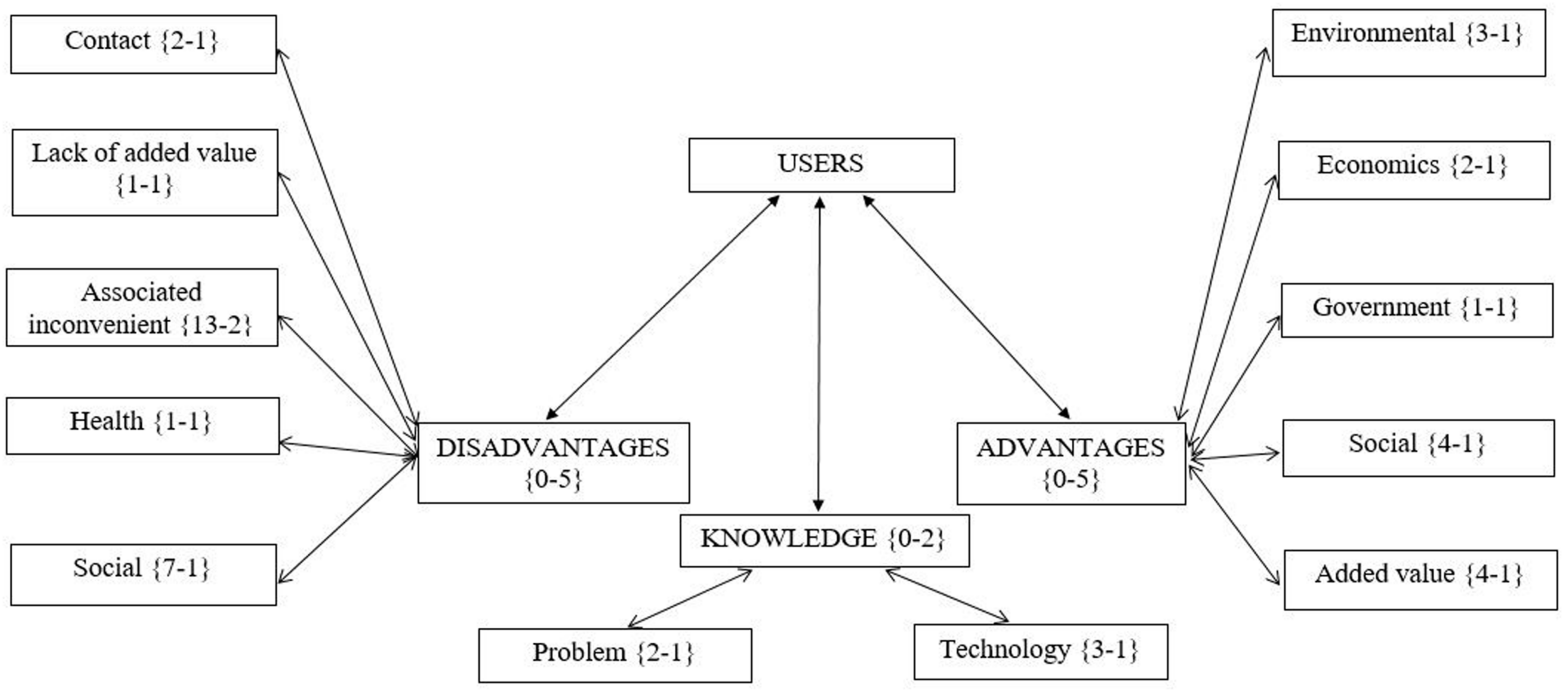
Qualitative analysis of the focus group at the business center.

Participants expressed psychosocial barriers similar to those observed in other contexts of acceptance of decentralized wastewater treatment systems (Mankad, 2012; Singh et al., 2014; Simha and Ganesapillai, 2017). Specifically, the qualitative analysis of the data resulted in the proposal of three central categories: (a) knowledge, (b) advantages, and (c) disadvantages (see Figure 1).

The “knowledge” category highlights two points of interest. On one hand, users do not consider there to be a water quantity or quality problem in the area. The lack of perception of a water problem that requires a solution leads users to view changing existing systems as unnecessary, resulting in greater resistance to decentralized plants (Gómez-Román et al., 2020; Nancarrow et al., 2010). On the other hand, participants referred to know the existence of the technology used in the decentralized plant of the building. However, since the plant became known due to the initial technical incident mentioned earlier, awareness of its existence could be related to high uncertainty (Yates and Stone, 1992).

Users articulated more disadvantages than advantages regarding the decentralized plant, revealing their discomfort with the technology, similar to what was found in other projects (Domènech and Saurí, 2010). They voiced concerns about the maintenance demands of the plant (Mankad, 2012; Mankad et al., 2010); but the primary drawback was the odors (Haddad et al., 2018), one of the consequences of the technical incident in the building’s plant. The emotional reaction of disgust associated with odors functions as a protective mechanism, prompting individuals to oppose the stimulus that is perceived as a pollutant, thereby increasing the rejection of the plant (Rozin et al., 2015).

The focus group also highlighted “social disadvantages.” Participants emphasized the general population’s (and building users) lack of preparation to undertake the necessary changes to implement these new technological systems (Moglia et al., 2011; Rygaard et al., 2014).

Despite the verbalized disadvantages, users also pointed out arguments in favor of the decentralized plant, mainly highlighting its added value compared to centralized plants: reduced operating costs and simplicity of system implementation (Ho and Anda, 2006). They also acknowledged the positive environmental impact the system can have. This suggests that the pro-environmental message has permeated the moral schemas of the population (Lewis et al., 2019; Poortinga et al., 2019). Despite the perceived environmental advantages, users indicated that social agreements are necessary before implementing decentralized systems. In this sense, they consider it crucial to emphasize the environmental contribution of the plant so that users feel part of the solution and express greater social support for the decentralized system (Gómez-Román et al., 2020; Mankad et al., 2010).

Given the existence of negative perceptions among users, partly attributable to the uncertainty produced by how they learned about the system (technical incident), Phase 2 proposes an intervention in which information is provided to improve attitudes toward the decentralized plant.

## 3 Phase 2. Priming environmental concern to improve acceptance

Providing specific information about the technology can help eliminate doubts and reservations that users may have about the plant. However, the mere provision of data may be insufficient. It is necessary to consider how that information is presented and integrated into the “common sense” or pre-existing social frameworks (Gramsci, 1971).

The results from the focus group indicate that users perceive one of the plant’s main strengths to be its positive environmental impact. In this sense, the activation and accessibility of environmental concerns can positively influence attitudes, emotions, and behaviors toward decentralized plants, thereby increasing their acceptance (Gómez-Román et al., 2021). This accessibility can be achieved through priming. By exposing users to information about well-known environmental problems, these concerns become more accessible, thereby affecting subsequent attitudes and decisions regarding the decentralized plant (Jonas and Sassenberg, 2006; Custers and Aarts, 2010; Scheufele and Tewksbury, 2007).

Our research not only focuses on activating environmental concerns through priming but also considers the impact of the unilaterality/bilaterality of the arguments presented about decentralized plants. Unilateral arguments highlight the advantages, while bilateral arguments provide a balanced view by including both positive and negative aspects of the technology.

The evidence on which type of argument—unilateral or bilateral—is more effective remains inconclusive, as several factors may influence its impact (Allen, 1991). Unilateral messages, which present only the positive aspects of an argument, tend to be more direct and effective, especially when the audience is unfamiliar with an issue. In contrast, bilateral messages (i.e., including advantages and acknowledging disadvantages) may reinforce the credibility of the source and increase confidence in the veracity of the message, if users already have a formed opinion on the issue. Thus, they can appreciate the complexity and honesty of acknowledging different points of view (Petty and Cacioppo, 1986).

In this regard, the objective of this phase is to understand the effect of environmental priming and the presentation of unilateral or bilateral information on the perception of business center workers regarding the decentralized wastewater treatment system (based on Gómez-Román et al., 2021). The main hypothesis is that environmental priming will positively influence the perception of the building’s decentralized plant. Additionally, public perception of decentralized plants is expected to be more favorable when only the advantages of these installations are presented. However, we anticipate a significant interaction between environmental priming and the type of information presented about the plants. Specifically, in the absence of environmental priming, public perception will be more negative when discussing disadvantages than when only advantages are presented. Conversely, in the condition with environmental priming, we expect the perception of decentralized plants to be similar regardless of whether advantages alone or both advantages and disadvantages are presented.

### 3.1 Materials and methods

#### 3.1.1 Participants and design

A total of 46 workers from the Porto de Molle building participated in this second phase (70.6% men, *M*_age_ = 41.06, *SD* = 10.24). The experimental design was a 2 (priming: environmental priming vs. company piracy) × 2 (information: advantages vs. advantages and disadvantages).

#### 3.1.2 Procedure

Recruitment was conducted by two researchers who approached workers in person, going door-to-door throughout the building to ask for their collaboration in a university-led research study. Participants were provided with a link to an online questionnaire on Qualtrics, which they could complete at their convenience on their own devices. The researchers were not present while the participants filled out the survey, ensuring privacy during the process. In the introductory section of the questionnaire, participants were informed about the confidentiality and anonymity of their responses, and the data protection policy was clearly outlined.

Participants were required to provide informed consent. Subsequently, each participant was randomly assigned to one of the experimental conditions. To prepare them for the procedure, participants were first given an example topic along with three questions to familiarize themselves with the format. This introductory step served as the initial priming manipulation. It was followed by the presentation of the informational text, which constituted the other experimental manipulation. Finally, participants were asked questions related to the installation of the decentralized plant.

##### 3.1.2.1 Environmental priming vs. company piracy

The environmental priming consisted of two phases to make the environmental problem accessible. First, the experimental group read a text about the consequences of climate change (see Supplementary material). Second, participants had to respond to the following statements: (a) “I consider these environmental problems to be…” (from 1 = *not serious at all* to 9 = *very serious*), (b) “Addressing these environmental problems is” (from 1 = *not urgent at all to* 9 = *very urgent*) and (c) “As a citizen, I should be more involved in solving these environmental problems” (from 1 = *totally disagree* to 9 = *totally agree*).

The control condition consisted of a text about piracy in companies. This topic was selected because it is neutral with respect to environmental issues, yet relevant to the business context and capable of engaging the interest of workers. By choosing a topic that could generate attention without introducing environmental biases, we ensured that any observed effects could be attributed specifically to the environmental priming, rather than general attitudes or engagement elicited by an unrelated, random topic. Similarly to experimental condition participants had to respond to three questions expressing their opinions on the severity, urgency of the problem, and citizen involvement.

##### 3.1.2.2 Advantages vs. advantages and disadvantages

Once the priming was introduced, participants were informed that they would be answering the research questions. A text about the wastewater treatment plant installed in the building’s basement was presented. In the “advantages” condition, only the advantages of the decentralized plant were provided, while in the “advantages and disadvantages” condition, both the advantages and disadvantages were presented (see Supplementary material for complete information).

##### 3.1.2.3 Priming control

At the end of the questionnaire, participants were asked to answer the following open-ended question: “What do you think is the objective of this research?” This statement allowed us to identify whether or not participants were aware of the experimental manipulation, and if they were, their responses were not included in the analysis (Bargh, 2006; Furnham, 1986). The review of the responses to the open-ended question suggests that no participant identified the manipulation or the study’s objective. Participants typically mentioned that the study aimed to understand their perceptions and opinions about the decision to install a plant in the building or to provide information about the existing water treatment. They did not explicitly or implicitly refer to the effect that the first task of the questionnaire (priming) might have on the second part of the study (acceptance of the decentralized plant).

#### 3.1.3 Measures

All the measures were adapted from the study of Gómez-Román et al. (2021).

##### 3.1.3.1 Attitudes toward decentralized plants

Attitudes were measured using a 9-point semantic differential scale consisting of eight items. Participants had to rate to what extent the installation of the decentralized plant in the building was: (a) *very bad* – *very good*, (b) *dislike it very much* – *like it very much*, (c) *very negative* – *very positive*, (d) *very unnecessary* – *very necessary*, (e) *very useless* – *very useful*, (f) *very unacceptable* – *very acceptable*, (g) *very inappropriate* – *very appropriate*, (h) *very harmful* – *very beneficial* (α = 0.97, ω = 0.97).

##### 3.1.3.2 Strength of attitudes

The measure consisted of three items in which participants were asked to respond about the opinions they had just given regarding the installation of the decentralized plant in the building: (a) “How convinced are you about your opinions on the decentralized plant?” (from 1 = *not at all convinced* to 9 = *very convinced*), (b) “How confident are you in your opinions about the decentralized plant?” (from 1 = *no confidence* to 9 = *a lot of confidence*), and (c) “If in a conversation someone disagreed with your opinion about the installation of the decentralized plant, do you think you would change your opinion?” (from 1 = *very easily* to 9 = *very difficultly*) (α = 0.91, ω = 0.91).

##### 3.1.3.3 Emotions

Participants were asked to indicate to what extent the installation of the plant in the building made them feel (from 1 = *nothing* to 9 = *a lot*): worried, disgusted, angry, fearful, helpless (negative emotions: α = 0.84, ω = 0.84); and relieved, proud, optimistic, enthusiastic, comfortable (positive emotions: α = 0.72, ω = 0.68).

##### 3.1.3.4 Behavioral intention

The measure consisted of five items in which participants were asked to respond to the following questions: (a) “If you had to choose between this decentralized plant in the building or the traditional centralized system used by the other buildings in Porto de Molle, would you choose the decentralized plant installed in the building?” (from 1 = *strongly disagree* to 9 = *strongly agree*), (b) “If you had been able to vote for the installation of this decentralized plant in the building, how would you have voted?” (from 1 = *totally against* to 9 = *totally in favor*), (c) “Would you have campaigned in favor of the installation of this decentralized plant in the building?,” (d) “Would you recommend installing decentralized plants in other buildings similar to the business center?,” and (e) “If you had the necessary financial resources, would you install a decentralized plant in the building/house where you live?.” Items *c*, *d*, and *e* were answered on a scale from 1 = *definitely not* to 9 = *definitely yes*. The internal consistency indices for the five items were: α = 0.90, ω = 0.89.

### 3.2 Results and discussion of phase 2

To evaluate whether environmental priming and information (unilateral and bilateral) improved workers’ perceptions of the building’s decentralized plant, we conducted an univariate analysis of variance for each dependent variable (Levene’s test for homogeneity was not significant, as detailed in Supplementary material). Table 1 shows the detailed results of the effects of environmental priming, information, and their interaction. The results show that participants in the environmental priming condition exhibited more positive attitudes, greater strength in their attitudes, more positive emotions, fewer negative emotions, and greater behavioral intention (similar to Gómez-Román et al., 2021). However, in this study with workers, the priming effect was significant only for attitudes (*F* = 5.89, *p* = 0.020, η^2^ = 0.123).

**Table 1:** Univariate analysis: simple effects and interaction of priming and information on acceptance.

Variable	Condition	Condition level		*n*	*M*	*SD*	*F*	*p*	η^2^
Attitudes	Priming	Control		23	7.42	1.40	5.89	0.020	0.123
		Environmental		23	8.23	0.81			
	Information	Advantages		25	8.03	1.28	1.93	0.172	0.044
		Advantages + disadvantages		21	7.59	1.09			
	Priming X Information	Control	Advantages	13	7.63	1.61	0.001	0.978	0.001
			Advantages + disadvantages	10	7.16	1.10			
		Environmental	Advantages	12	8.46	0.60			
			Advantages + disadvantages	11	7.98	0.96			
Attitudes strength	Priming	Control		23	6.97	1.21	1.38	0.246	0.032
		Environmental		23	7.35	1.43			
	Information	Advantages		25	7.56	1.22	5.73	0.021	0.120
		Advantages + disadvantages		21	6.68	1.32			
	Priming × information	Control	Advantages	13	7.49	1.16	0.595	0.445	0.014
			Advantages + disadvantages	10	6.30	0.95			
		Environmental	Advantages	12	7.64	1.32			
			Advantages + disadvantages	11	7.03	1.55			
Negative emotions	Priming	Control		23	3.14	1.50	1.88	0.178	0.043
		Environmental		23	2.49	1.62			
	Information	Advantages		25	2.78	1.78	0.038	0.847	0.001
		Advantages + disadvantages		21	2.85	1.34			
	Priming × information	Control	Advantages	13	3.14	1.75	0.036	0.850	0.001
			Advantages + disadvantages	10	3.14	1.18			
		Environmental	Advantages	12	2.40	1.81			
			Advantages + disadvantages	11	2.58	1.48			
Positive emotions	Priming	Control		23	5.71	1.30	3.50	0.068	0.077
		Environmental		23	6.35	0.93			
	Information	Advantages		25	6.08	1.27	0.121	0.730	0.003
		Advantages + disadvantages		21	5.98	1.05			
	Priming × information	Control	Advantages	13	5.77	1.38	0.001	0.977	0.001
			Advantages + disadvantages	10	5.64	1.26			
		Environmental	Advantages	12	6.40	1.11			
			Advantages + disadvantages	11	6.29	0.73			
Behavioural intention	Priming	Control		23	7.04	1.75	0.577	0.452	0.014
		Environmental		23	7.41	1.35			
	Information	Advantages		25	7.33	1.74	0.278	0.601	0.007
		Advantages + disadvantages		21	7.10	1.33			
	Priming × information	Control	Advantages	13	7.00	2.12	0.486	0.489	0.011
			Advantages + disadvantages	10	7.08	1.21			
		Environmental	Advantages	12	7.68	1.19			
			Advantages + disadvantages	11	7.11	1.50			

Regarding the information (unilateral vs. bilateral), participants who read both the advantages and disadvantages of the plant showed fewer positive attitudes, less strength in these attitudes, fewer positive emotions, more negative emotions, and less behavioral intention. However, the effect was significant only for the strength of attitudes (*F* = 5.73, *p* = 0.021, η^2^ = 0.120). These findings contrast with the results found in hypothetical contexts, where presenting the disadvantages alongside the advantages results in less acceptance overall (Gómez-Román et al., 2021). No significant interaction between environmental priming and the information provided is found in the case study context either.

In summary, although the observed trends among workers point to their greater disposition toward the plant when only advantages are provided in the environmental priming condition, most of the effects are not significant. There are three reasons that may explain these findings. First, although the number of participants is considerable given the total number of workers in the building, it is still a small sample size. Second, the life stage and characteristics of workers in a business center are different from those samples commonly used in experimental studies, such as students, that do find significant effects (Gómez-Román et al., 2021). In this sense, and at least in the specific case of decentralized plants, we believe it is necessary to be cautious about generalizing the results of student samples to the general population (Hanel and Vione, 2016). Third, in contrast to hypothetical situations, in the business center where the plant was already installed, users had experienced certain associated problems. In each context, the personal relevance of the plant to the participants is different; therefore, the degree of information permeability in users’ attitudes is also expected to vary.

Exploring these trends in a real context contributes to the knowledge base on the social acceptance of decentralized wastewater treatment plants. It highlights the importance of considering the applicability of results according to the context. Overall, the results emphasize the importance of considering how content is presented. The first contact with information about a new technology establishes an interpretative framework that guides the formation of perceptions and decision-making (Rogers, 2003), and the results suggest that environmental priming remains an element that promotes favorable attitudes toward decentralized treatment plants, even among workers who initially expressed reservations about the installed plant.

## 4 Phase 3. A bidirectional information session with experts to improve acceptance

The results of the previous phase indicate that making environmental issues accessible through priming improves workers’ attitudes toward decentralized treatment plants. However, it’s important to note that priming capacity is limited in fostering positive emotions, reducing negative emotions, and increasing behavioral intention. In contexts with high uncertainty, lack of knowledge, and novelty, such as the case study (due to problems arising from technical failure), protection mechanisms against potential threats are activated (Albers, 2012; Slovic et al., 2004). In this sense, the mere association of the plant as a solution to an environmental problem may not be sufficient to modify emotional variables, such as fear, and behavioral variables, such as verbalizing discomfort, associated with decentralized plants. Therefore, adequately communicating the procedures, benefits, and risks of the decentralized plant can help improve understanding (Fischhoff et al., 1993). In this way, the information could facilitate a change in risk perceptions and associated negative emotional reactions (Vila-Tojo et al., 2024). However, while the content of the information is relevant, its interpretation by the public relies more on who and how the information is presented (Entman, 1993).

Trust in the source is key in forming positive or negative perceptions (Ryu et al., 2018). The observable and inferred characteristics of the source, such as competence and intentionality, are crucial for the message’s effectiveness (Milburn, 1991; Perloff, 1993; Twyman et al., 2008). In this context, experts, especially scientists, are perceived as credible and trustworthy sources (Cologna and Siegrist, 2020), making them particularly persuasive (Meyer, 1988). While competence-based trust is relevant, intentionality-based trust, linked to the source’s honesty and integrity, has a greater influence on the acceptance of new technologies (Liu et al., 2020). This underscores the importance of selecting communicators who possess the necessary technical knowledge and are seen as authoritative and trustworthy sources.

In addition to the characteristics of the source, interactive communication and the opportunity for dialogue are important for building trust and credibility. Allowing questions and encouraging discussions creates a more engaging and trustworthy environment (Brashers, 2001). Research shows that interactive participation significantly improves acceptance of new technologies by addressing concerns and providing real-time clarification (Van den Hooff and De Ridder, 2004; Venkatesh et al., 2003).

Consequently, the third phase of the intervention seeks to establish a bidirectional communicative environment aimed at improving users’ perceptions of the decentralized plant. On the one hand, the high reliable source emphasizes environmental issues and presents information about the plant. On the other hand, users can address their doubts and discuss on topics related to the decentralized plant. With these premises, an informative session (workshop) was designed for all Porto de Molle workers who wished to attend.

### 4.1 Materials and methods

#### 4.1.1 Participants

In the workshop, 25 workers from the business center participated. However, not all of them agreed to complete the questionnaire. Consequently, the final sample consisted of 20 participants (65% men, *M*_age_ = 40.56, *SD* = 9.11). Most of the participants had been working in the building for less than 1 year (*less than 6 months* = 20%, *between 6 months and 1 year* = 40%, *between 1 and 2 years* = 10%, *more than 2 years* = 30%).

#### 4.1.2 Procedure

The workshop took place in the business center building. The building’s management sent an email invitation to participate in the session, indicating that the workshop would provide information about the new decentralized plant in the building. A pre-post test intervention was conducted. First, the baseline perception of the decentralized plant installed in the building was measured. Data was collected via a questionnaire before the workshop began. These questions were also asked at the end of the session to find out if there had been a change in attitudes.

The data collection process was structured, with the pre-test questionnaire divided into three parts: an introduction, information about the plant, and questions about its acceptance. The post-test questionnaire, administered after the workshop, was similar to the pre-test, allowing participants to reevaluate their responses and provide additional sociodemographic data.

A technical professional attended the session, providing information on the technology and operation of the decentralized plant. A social psychologist also took part in the session, going in-depth on the aspects of sustainability linked to the implementation of this new technology. At the end of the presentations, participants ask questions and discuss with the experts about the information received.

#### 4.1.3 Measures

##### 4.1.3.1 Attitudes toward decentralized plants

Attitudes were measured using a 9-point semantic differential scale consisting of three items. Participants had to rate to what extent the installation of the decentralized plant in the building was: (a) *very bad – very good*, (b) *very unnecessary – very necessary*, (c) *very unacceptable – very acceptable* (α = 0.92, ω = 0.92).

##### 4.1.3.2 Emotions

Participants were asked to indicate to what extent the installation of the plant in the building made them feel (from 1 = *nothing* to 9 = *a lot*): worried, disgusted, (negative emotions: *r* = 0.764, *p* < 0.001); and relieved, proud (positive emotions: *r* = 0.824, *p* < 0.001).

##### 4.1.3.3 Behavioral intention

Participants were required to respond to items *a* and *d* from phase 2. That is, whether they would choose the decentralized plant installed in the building (from 1 = *strongly disagree* to 9 = *strongly agree*) and if they would recommend installing the decentralized plant in other buildings with similar characteristics to the business center (from 1 = *definitely not* to 9 = *definitely yes*) (*r* = 0.849, *p* < 0.001).

##### 4.1.3.4 Change of opinion

In order to find out whether the participants recognized a change of opinion in their attitudes after receiving the information, we asked them whether they considered that their opinion of the decentralized plant had changed after the information session (dichotomous: *no* or *yes*). If so, they were asked to indicate whether their opinion had improved or worsened (from 1 = *much worse* to 9 = *much better*). Finally, they were asked to answer the following open-ended question: “If you have changed your opinion, what arguments/information have caused this change?”

### 4.2 Results and discussion of phase 3

The study’s objective was to examine whether a bidirectional informative session about the operation of the decentralized plant and its added value as a solution to an environmental problem could improve users’ perception of the decentralized plant installed in their building.

The results indicate that the workshop significantly affected the perception of the plant (see Table 2). Specifically, the participants showed a more favorable attitude toward the plant, a reduction in negative emotions, and an increase in both positive emotions and behavioral intention. In this sense, the findings indicate that providing detailed and contextualized information, along with opportunities for interactive participation, significantly improves technology acceptance (Brashers, 2001; Hou et al., 2021; Ranney and Clark, 2016; Taube et al., 2021).

**Table 2:** Mean differences between pre-test and post-test on the acceptance variables.

Variable	Pre-test		Post-test		*t*	*p*	Cohen’s *d*
	*M*	*SD*	*M*	*SD*			
Attitudes	6.28	0.89	7.90	0.71	−6.34	<0.001	0.81
Negative emotions	5.45	1.28	2.42	1.37	7.23	<0.001	1.32
Positive emotions	5.05	1.41	7.25	1.25	−5.21	<0.001	1.34
Behavioural intention	6.00	1.27	7.83	0.89	−5.27	<0.001	1.10

Overall, the participants stated that their opinion of the decentralized wastewater treatment plant had improved (*M* = 7.40, *DT* = 1.18). Among the reasons participants cited for their change in opinion, they highlighted the positive environmental impact of the technology, including water savings, nutrient recovery, and environmental value. Additionally, they emphasized the positive impression generated by the technical information, practical examples, and the novelty of the technology. Specifically, the reasons provided by participants suggest that not all types of information are effective. In particular, scientific-technical information communicated by reliable experts allows users to understand the benefits and operation of the plant and to resolve the discomfort and uncertainty associated with it (Liu et al., 2020).

However, a limitation of this study is the low participation in the workshop, with only 10% of the building’s workers attending. Additionally, we do not control for possible overlap between participants across phases. This means that some individuals may have been exposed to the different phases, potentially influencing their responses. Despite this, the consistent positive effects observed in Phase 3 suggest that the workshop was effective, either on its own or through an accumulated effect from exposure across multiple phases. Moreover, since the evaluation of attitudes in Phase 3 was conducted immediately after the intervention, we do not know the stability of this change in perception in the long term. As a consideration for future studies, conducting follow-ups at different times after the intervention would be interesting to verify whether the positive attitude toward the technology is maintained over time and explore strategies to increase participation in these informational events.

## 5 General discussion and conclusion

Society faces an urgent climate challenge to which we must respond (Wyss et al., 2021). Although technological solutions have already tried to address environmental problems, we should remember that their implementation is conditional on the support received at the political and social level (Dimitrov, 2020; Lahsen and Turnhout, 2021). Decentralized wastewater treatment plants do not always have such citizen support. Overcoming the citizen reluctance requires the design of intervention strategies that consider the barriers detected in each specific context. In this study, we present an intervention carried out in a business center, which is of particular interest due to the discomfort generated by a technical failure in installing the decentralized wastewater treatment plant (of whose existence users were unaware). In this intervention, uncertainty was addressed through the mitigation of the associated epistemic demand (Sabucedo et al., 2020). In new and unknown situations, people need information that allows them to position themselves and make decisions.

This scenario led us to propose a first phase that consists of a focus group that would allow us to identify the main barriers associated with the plant and initiate a communication channel with users. The results showed a negative perception of the plant similar to works in other contexts (Koetse, 2005; Mankad and Tapsuwan, 2011; Rygaard et al., 2014). The participants’ verbalization of more disadvantages than advantages allow for three observations. First, there is a strong psychological distance (Keller et al., 2022). Users recognize the value of the decentralized plant in solving environmental problems. However, at the same time, they do not perceive a problem in the area, making the plant seem unnecessary to them. Second, the opinion about the cost–benefit of the plant is not uniform; users verbalize concern about the cost of maintaining the plant while recognizing its low operating cost and ease of installation (Ho and Anda, 2006). Third, the “yuck factor” is one of the main variables associated with users’ discomfort, related to concerns about the smell and color of the water (Rozin et al., 2015; Wester et al., 2016).

With these observations, we proposed a second phase to activate environmental concerns and provide information about the plant’s advantages and disadvantages that would alleviate discomfort and uncertainty. The findings showed that activating environmental concern (through priming) is related with higher attitudes toward the technology. However, priming effect on negative emotions, positive emotions and behavioral intention was not significant. In this regard, it is important to consider the ongoing debate about the replicability of priming experiments (Chivers, 2019; Rohrer et al., 2019).

Therefore, in the third phase, we opted to design a workshop based on trust in the source and focused on informing about the plant’s properties and its capacity to solve a concerning environmental problem. The perception and emotions associated with the plant improved significantly after the informational session. This change underscores how framing is an important element for persuasion (Benford and Snow, 2000), especially in contexts of uncertainty, such as the case at hand. In such situations, people are more receptive to messages that alleviate their uncertainty, especially if they come from reference groups (Schultz and Fielding, 2014). In this sense, the workshop results showed, as has been pointed out many times in the literature, the significant role of scientists as a source of trust for environmental issues (Cologna and Siegrist, 2020). Due to their training and experience, they are seen as reliable and objective experts, increasing public receptivity to the information presented (Meyer, 1988). However, we must remember that they also need to align the presented information with the real expectations and concerns of the public (Sawyer and Ball, 1981). This matter is particularly relevant because scientists are not the only reference group for users. Other trusted agents could capitalize on discontent and channel uncertainty toward positions and actions contrary to the decentralized system. In future studies, it would be advisable to explore the effect of the interaction of different sources of influence on the acceptance of plants. For example, the intervention was coordinated by university profiles that generally receive positive public evaluations. Such an academic context could have influenced the favorable response of the building users (Sanz-Menéndez and Cruz-Castro, 2017).

Alongside the contributions of the use of reliable sources to transmit technical and environmental information, this third phase also emphasizes the relevance of active user participation to alleviate uncertainty. Receiving information passively can have a reduced effect if it is not accompanied by other techniques (Schultz, 1998). In contrast, interactive interventions allow for the expression of opinions and provide spaces for discussion to foster acceptance. On the one hand, they contribute to the decision not being perceived as externally imposed. On the other hand, they increase commitment to the decisions made (Lewin, 1943; Jans, 2021).

The intervention presented in this work indicates that the acceptance of decentralized plants, as with other behaviors, depends on the beliefs developed during social interaction (Jaspal and Breakwell, 2014). Therefore, it is important to incorporate (potential) users throughout the entire process, from beginning to end, with the aim of weaving complicity between promoters and users and generating a climate of co-responsibility regarding the plant. This participatory approach helps to prevent the emergence of psychosocial resistance and can also alleviate existing doubts and misgivings about the operation of decentralized plants. Engaging community members from the outset fosters ownership, making them more likely to support the adoption of such technologies, as they feel heard and actively involved in the decision-making process.

While this study focused on a business center, the intervention protocol can be adapted to other settings, such as residential areas or different cultural contexts. For example, in residential communities, the focus group phase could be adapted into neighborhood meetings to address local concerns, and the workshop could include more context-specific examples that make the benefits of decentralized systems relatable. Implementing this protocol in diverse cultural settings would also require careful attention to local norms, trust in authorities, and perceptions of water reuse (Vila-Tojo et al., 2022). Tailoring the intervention to address these factors would enhance acceptance, making the findings more generalizable across different contexts.

## Data Availability

The raw data supporting the conclusions of this article will be made available by the authors without undue reservation.
