# Supplementary material for: The role of information and participation in overcoming users’ initial reluctance: a case study of a decentralized wastewater treatment plant
Source: Front Psychol. 2024 Nov 5;15:1445320. doi: 10.3389/fpsyg.2024.1445320 (PMC11573550; doi:10.3389/fpsyg.2024.1445320)
Supplement: Supplementary file 1 [file Data_Sheet_1.docx]

**SUPPLEMENTARY MATERIALS**

**Phase 1. Focus Group**

**Information provided to participants at the introduction of the session**

Good morning/afternoon.

We are from the University of Santiago de Compostela, and we are conducting a research project on specific aspects of environmental sustainability. This study is funded by the European Commission.

Thank you for joining us and dedicating approximately 60-90 minutes of your time. Firstly, please complete an informed consent form. This form requires your personal information and your signature to authorize the recording of this conversation. You may withdraw your participation at any time. We assure you that the information you provide will remain anonymous. All data will be analyzed collectively and not on an individual basis.

During our session, we will present various questions and open the floor for discussion on each topic. You are encouraged to express your opinions freely. Participants may agree, disagree, elaborate on points, comment, or reflect. Think of it as a friendly chat. Our role is to facilitate the discussion, for example, by introducing new questions when a topic is thoroughly discussed or gently steering the conversation back if it strays off-topic.

**Informed Consent Form**

Date: __ / __ / __

Start Time: ________ End Time: ________

his interview aims to gather your opinions on environmental and sustainability issues at the Porto de Molle industrial park. For professional and ethical reasons, it is important to note that your participation is entirely voluntary, and you are free to withdraw at any time. If you choose to participate, we ask for your consent to audio-record the interview. This helps us to more accurately capture and remember the information you provide.

I agree: _______________________________________

**Focus Group Script**

| **GUIDING CATEGORIES** | **INVESTIGATION AXES** | **GUIDING QUESTIONS** |
| --- | --- | --- |
| **CULTURAL LEVEL** | Ideology related to the environment. | 1. What environmental problems are most urgent in the Porto de Molle industrial park? *If they mention water, ask why*. |
|  | Ideology related to water management priorities (*Ask if not mentioned in Q.1*). | 1. What environmental problems are most urgent in the Porto de Molle industrial park? *If they mention water, ask why*. |
|  | Knowledge and opinion on circular economy. | 1. How much do you know about circular economy approaches: water reuse, nutrient recovery from the source, etc.? What is your opinion? |
|  | Knowledge and opinion on the system used in the building. | 1. How do you assess the impact of new environmental technologies (purification, water recycling) for environmental improvement and care? |
|  | Knowledge and opinion on the system used in the building.  (*If in the previous question participants do not mention that the building uses R4L technology, ask the following question*). | 1. Did you know the building has an installed water purification system that separates waste from the source and treats it differently to reuse and promote the circular economy? |
| (INTER)PERSONAL LEVEL | Perception. | 1. What do you think about the system? Do you like it? |
|  | Perception. | 1. On a scale of 0 to 10, how would you rate the overall water sanitation management of the building? Why? |
|  | Perception of benefits. | 1. What positive aspects would you highlight about this water treatment system in the building? What do you think are the advantages/benefits of this system? |
|  | Perception of risk. | 1. What potential problems might its operation have? What would you say the disadvantages are? |

**Data Analysis Method**

**Phase 1. Coding:** We applied an integrated approach for thematic analysis (Cruzes & Dyba, 2011) that employs a deductive approach (Miles et al., 1994) to create semantic domains and an inductive approach (grounded theory) (Corbin & Strauss, 2008) for the creation of codes. Researcher 1 creates a list of semantic domains where the codes are subsequently grouped inductively. These initial domains integrate concepts known in the literature. These codes must meet two requirements that Atlas.ti defines as follows (ATLAS.ti, 2019): (1) *exhaustiveness*, meaning the codes in the codebook must cover the variability in the data, and no aspect relevant to the research question should be left out. (2) *mutual exclusivity*, meaning (i) the codes within each domain must be different and this must be specified in the code definitions, and (ii) at most one of the codes in a semantic domain can be applied to a quote or overlapping quotes. So, the codes must have explicit boundaries so they are not interchangeable or redundant. To avoid bias and ensure that the codes mean the same to anyone using them.

Next, two researchers separately approached the data (i.e., the focus group transcription) with these codes in mind. The data were reviewed line by line, citations (text segments) were created, and the previously defined codes were assigned where appropriate.

**Phase 2. Improving coding reliability:** Intercoder Agreement (ICA) analysis techniques were used to test the reliability of the results. Coder 1 and Coder 2 participated in coding the interview transcripts using the codebook. This phase was carried out as follows: Krippendorff's α coefficients were applied after coding. If the coders did not reach an acceptable level of reliability, the disagreements were analyzed to determine why Coder 1 and Coder 2 did not understand a code in the same way, making the necessary adjustments to the codebook. Then, both coders performed a new coding. This process should be repeated, if necessary until an acceptable level of reliability is reached. Table 1 shows the level of reliability in the first coding and Krippendorff's α coefficients in the second, when an adequate level of agreement was considered to have been reached. Generally, α coefficients range from 0 ≤ α ≤ 1. A common empirical rule in the literature (Krippendorff, 2018) is that Cu α ≥ 0.667 is the minimum threshold required to draw conclusions from the data. For Cu α ≥ 0.80, we can consider that there is statistical evidence of reliability in the assessments (Díaz et al., 2021).

**Table 1. Reliability coefficient values in the inter-rater comparison, comparison between the first and second reviews.**

| **Coefficient** | **Semantic Domain** | **Round 1** | **Round 2** |
| --- | --- | --- | --- |
| **% Simple Agreement**  (Calculated as the number of times a set of ratings is the same, divided by the total number of observation units that are rated, multiplied by 100). | Knowledge | 11% | 49.8% |
|  | Advantages | 13.6% | 63.3% |
|  | Disadvantages | 13.5% | 72.6% |
|  | Total | 14.3% | 73.6% |
| **α Binary**  (It is a measure of the degree of agreement to which coders choose to apply or not apply a semantic domain). | Knowledge | .181 | .646 |
|  | Advantages | .244 | .755 |
|  | Disadvantages | .323 | .837 |
|  | Total | .256 | .725 |
| **Cu-α**  (It is a global measure of the goodness of fit in semantic domains. It measures the degree of reliability in the decision to apply the different semantic domains, regardless of the code chosen.) |  | .377 | .919 |

**Phase 3. Synthesis:** Once an adequate level of agreement was reached and the codes were ideally assigned to quotations, the relevant analyses were performed to extract the results.

**Definition of Codes**

**Knowledge**

Knowledge: This section includes two codes that aim to group all ideas/opinions related to the level of knowledge about the issues addressed by decentralized treatment plants, such as technical aspects related to their operation or the technical aspects of the products obtained from recovery (water and fertilizers).

- **c_problem**: Refers to environmental issues related to water reuse, nutrient deficiency, sustainability problems, pollution, climate change, etc.
- **c_technology**: Refers to technical aspects of the system, which can be elements related to technology or aspects of chemical nutrients, etc.

**Disadvantages**

This section includes all codes that group perceived disadvantages, difficulties, or barriers associated with implementing both the technology and the products obtained.

- **d_social**: Refers negatively to the general population, future users, etc. Issues related to the rejection of the end-users of the technology or the lack of demand or awareness of the need.
- **d_contact**: Refers to elements of disgust or discomfort due to high contact with waste by the users.
- **d_ecological**: Environmental impact that the technology itself may have.
- **d_economic**: Refers to economic aspects linked to implementation. Additional installation costs, maintenance costs, necessary investment for implementation, etc.
- **d_governance**: Elements related negatively to political will. Issues of policymakers and political decisions that may be an impediment.
- **d_associated_inconveniences**: Any other issue that may be referred to which has not been covered in the above points. Here, it can include visual impact, odors, space, maintenance, misuse of the system by users, etc.
- **d_legal**: Lack of laws and regulations that facilitate implementation.
- **d_health**: References to the consequences of reuse on public health, whether it be from water reuse or consuming products produced with recovered nutrients. Health-related concerns.
- **d_lack_of_added_value**: Comments and opinions regarding the low contribution of the technology or the recovered resources (“There were already other strategies that worked”).

**Advantages**

This category of codes is categorized as perceived advantages or facilitators of implementing both the technology and the products obtained.

- **v_social**: People will support the implementation/use of the technology and the derived products. Issues related to the acceptance of the technology by the end-users or the perceived need by the population for its use.
- **v_contact**: The fact that it involves low contact can be a facilitator.
- **v_ecological**: It supports mitigating climate change or is more sustainable than other alternatives.
- **v_economic**: The cost is lower than that of current systems in the short or long term. References to potential cost savings.
- **v_legal**: References to legal/regulatory aspects that may favor its implementation.
- **v_governance**: Elements related positively to political will. Issues of policymakers and political decisions that may be facilitators.
- **v_added_value**: Comments and opinions regarding the contribution of the technology or the recovered resources compared to what is already implemented or in operation. The innovative component. Elements of pride or satisfaction for using something new, different from what was previously used, or for doing something positive to mitigate climate change.

**Phase 2. Environmental Concern Priming Experiment Texts**

**Company Piracy Condition**

Before you begin the questionnaire for this study, we will introduce a scenario accompanied by three questions to help you familiarize yourself with the procedure. Please read the text carefully and respond to the questions sincerely. Once you are clear on how to proceed and have no further questions, we will move on to the main questionnaire.

**The effects of piracy**

In recent years, major audiovisual media companies have initiated legal actions and launched information campaigns against what they term "piracy." Data indicates that 25% of illegal content consumers believe that pirating movies or series does not harm anyone or any industry. However, industry leaders argue that piracy inflicts damage at three significant levels: it leads to a direct loss of profits, hinders the creation of numerous jobs, and results in lower public revenue compared to what would be collected from legal consumption.

**Environmental Priming Condition**

Before you begin the questionnaire for this study, we will introduce a scenario accompanied by three questions to help you familiarize yourself with the procedure. Please read the text carefully and respond to the questions sincerely. Once you are clear on how to proceed and have no further questions, we will move on to the main questionnaire.

**The Consequences of Climate Change**

The Intergovernmental Panel on Climate Change (IPCC) of the United Nations (https://archive.ipcc.ch/) periodically prepares assessment reports to disseminate available scientific and technical knowledge on climate change.

Some forecasts for the coming years include:

For ecosystems, an increase of between 1.5 and 2.5°C in the global average temperature, above pre-industrial levels, will represent significant risks for many unique and threatened systems.

Changes in precipitation and temperature also affect water availability. Areas affected by drought will expand, posing a threat to food production, water and energy supply, and health and increasing regional water demands for irrigation.

**Information only advantages**

In the other buildings in Porto do Molle, wastewater is channeled through sewer networks to the treatment plant in Nigrán, about 2 kilometers away. In contrast, this building (Business Center) has a **decentralized plant installed in the garage for wastewater treatment.**

This plant allows the wastewater generated in the building to be treated right here. This means that the different types of water—grey water (from sinks) and black water (from toilets)—generated in the building are collected separately and, once treated in the basement plant, are used for various purposes, such as filling toilet tanks or irrigating green areas.

This allows for a significant **saving of potable water**. Each toilet flush uses between 4 and 6 liters of potable water. Considering the number of people working in the building, this **saves about 4,600 liters** of potable water daily, which is very significant from an environmental standpoint.

Another advantage is that this plant allows for the **recovery of phosphorus** in the wastewater and its use as fertilizer. Phosphorus is a scarce mineral and has, therefore, become a strategic priority globally for food production.

**Information advantages + disadvantages**

In the other buildings in Porto do Molle, wastewater is channeled through sewer networks to the treatment plant in Nigrán, about 2 kilometers away. In contrast, this building (Business Center) has a **decentralized plant installed in the garage for wastewater treatment.**

This plant allows the wastewater generated in the building to be treated right here. This means that the different types of water—grey water (from sinks) and black water (from toilets)—generated in the building are collected separately and, once treated in the basement plant, are used for various purposes, such as filling toilet tanks or irrigating green areas.

This allows for a significant **saving of potable water**. Each toilet flush uses between 4 and 6 liters of potable water. Considering the number of people working in the building, this **saves about 4,600 liters** of potable water daily, which is very significant from an environmental standpoint.

Another advantage is that this plant allows for the **recovery of phosphorus** in the wastewater and its use as fertilizer. Phosphorus is a scarce mineral and has, therefore, become a strategic priority globally for food production. However, this plant also has some disadvantages. One is that, at certain times, malfunctions or maintenance involving opening tanks or septic pits can produce **unpleasant odors**.

Another disadvantage is that the **operation and maintenance costs** are higher than the current alternative of discharging grey and black water into the sewers.

Table 1. Results of the Levene´s Test of Homogeneity of Variance

| Variable | *F* | *df*1 | *df*2 | *p* |
| --- | --- | --- | --- | --- |
| Attitudes | .972 | 3 | 42 | .415 |
| Attitude Strength | 1.960 | 3 | 42 | .135 |
| Negative emotions | 1.136 | 3 | 42 | .345 |
| Positive Emotions | .228 | 3 | 42 | .877 |
| Behavioural Intention | .980 | 3 | 42 | .412 |

*Note.* The statistics were calculated based on the mean.
